# Amino Acid Composition of Meat from Smena 9 Broiler Chickens During Ontogeny Under a High Stocking Density and Dietary Adaptogens Complex

**DOI:** 10.3390/ani16132047

**Published:** 2026-07-03

**Authors:** Nadezhda V. Bogolyubova, Roman V. Nekrasov, Julia A. Bogolyubova, Nikita S. Kolesnik, Pavel D. Lakhonin

**Affiliations:** 1 Federal Research Center for Animal Husbandry Named After Academy Member L.K. Ernst, Dubrovitsy, Podolsk 142132, Russia; bogolyubovajulia@gmail.com (J.A.B.); kominisiko@mail.ru (N.S.K.); lakhonin.99@mail.ru (P.D.L.); 2 All-Russia Research Institute of Animal Physiology, Biochemistry and Nutrition—Branch of the Federal Science Center for Animal Husbandry Named After Academy Member L. K. Ernst, Borovsk 249013, Russia; nek_roman@mail.ru

**Keywords:** broiler chickens, amino acids, meat, breast, thigh, age, adaptogens, sex

## Abstract

Broiler meat is an important source of protein with a balanced essential amino acid profile; however, the environmental stress associated with a high stocking density can reduce its nutritional value. Adaptogen supplementation represents a promising strategy to mitigate this negative impact. This study aimed to evaluate the amino acid composition of the breast and thigh muscles of Smena-9 broiler chickens under a high stocking density and dietary supplementation with an adaptogens complex (dihydroquercetin + vitamins E and C). The experiment was conducted on 120 broilers allocated to four groups: recommended stocking density (negative control), high stocking density (positive control), high stocking density with adaptogens from day 21, and high stocking density with adaptogens from day 1 of the experiment. The stocking density changed as the bird grew. On day 21, the bird stocking density in the S(-)CON group was 38 heads per 1 m^2^, in the other groups it was increased by 10% and amounted to 42 heads per 1 m^2^. Each week, the stocking density changed and amounted to 32 and 35 heads per 1 m^2^ in 28–35 days, 21 and 23 heads per 1 m^2^ in 35–42 days, and 17 and 19 heads per 1 m^2^ in 43 days and until the end of fattening, respectively. The amino acid profile was determined by ion-exchange chromatography with post-column derivatization using ninhydrin. Age was the primary factor influencing the amino acid composition, followed by muscle type and stocking density. Sex-related effects were less pronounced and were observed at 24 days of age. Breast meat exhibited higher amino acid content than thigh meat (82.4–89.9 vs. 65,8–77.8 g/100 g dry matter for total amino acids, respectively). Under a high stocking density, the most notable changes were observed in the thigh muscle at early ages. By day 52, the birds receiving the adaptogen complex from day 1 showed an increase in the sum of essential amino acids in the breast meat (+2.4%, *p* < 0.05), with the most pronounced improvements observed for methionine (+5.9%, *p* < 0.001) and lysine (+3.9%, *p* < 0.05) compared with the recommended-density control group (S(-)CON). Additionally, early adaptogen administration preserved the levels of flavor-related amino acids between days 34 and 52, whereas a decline was observed in other groups, indicating maintained sensory quality with age. Thus, the early administration of the adaptogen complex appears to mitigate for high stocking density negative effects and contributes to preserving the nutritional value of broiler meat, although the underlying mechanisms remain hypothetical and require further.

## Simple Summary

Chicken meat is one of the main sources of complete protein in the human diet, containing a balanced set of essential amino acids. Under intensive rearing conditions, poultry are often exposed to various stressors, which can reduce the nutritional value of meat. The aim of this study was to evaluate the amino acid composition of meat from Smena-9 broiler chickens under a high stocking density and dietary supplementation with an adaptogens complex comprising dihydroquercetin, vitamins E and C. The objectives included studying the amino acid composition of muscle tissue at 24, 34, and 52 days of age and its dependence on sex. Age exerted the greatest influence on the amino acid composition, whereas sex exerted the least. The breast muscle is richer in amino acids than the thigh muscle. Using the adaptogen complex, especially from the first day of rearing, contributed to the preservation of high levels of essential amino acids in meat by the end of the rearing period. These findings provide a basis for improving broiler rearing technologies. This will help maintain the high quality and nutritional value of chicken meat even under intensive production methods, thereby providing consumers with a healthy and wholesome product.

## 1. Introduction

Poultry meat plays a significant role in the global production of animal protein. It accounts for approximately 40% of total meat consumption worldwide [1,2]. Chicken meat has a high protein content: approximately 23–25% in the fillet and 18% in the thigh [3]. Poultry proteins contain a wide range of amino acids, the most abundant of which are lysine (~8.7%), leucine (~7.8%), isoleucine (~3.6%), and valine (~4.8%), along with glutamine, asparagine, arginine, and alanine [4]. It is an affordable source of protein with a balanced composition of essential amino acids (EAA). Studies have shown that broiler chicken protein contains a higher amount of essential amino acids (up to 92%) than other types of meat (pork—88%, lamb—73%, beef—72%) [5]. The amino acid composition is an indicator of the nutritional value of meat. Amino acids also contribute to flavor. For example, the Maillard reaction allows free amino acids in cells to form aromatic compounds [6,7]. Free amino acids are among the main aromatic compounds in meat, imparting sour, sweet, bitter, salty, and other flavors. For example, glutamic acid (GLU) and aspartic acid (ASP) have a mixture of sour and meaty flavors. Serine, glycine, alanine, and others have a sweet flavor. Histidine, valine, arginine, and others have a bitter flavor. It has also been shown that certain combinations of amino acids determine meat flavor to a greater extent than individual amino acids [8].

The composition and content of amino acids in poultry proteins fluctuate. Many factors influence the composition and concentration of amino acids in meat, including breed, age, diet, sex, and others [9,10,11]. Studies of poultry meat composition have shown that meat quality changes with age and sex [12]. Therefore, research into the optimal slaughter age and sex of broiler chickens is essential for producing competitive meat with a rich amino acid composition and excellent palatability.

Poultry housing conditions can also influence the amino acid levels in muscle tissue [13]. Despite advances in animal welfare practices, rapidly growing modern commercial broilers are still exposed to environmental and housing-related stressors, including stocking density, microclimate, and lighting regimes [14]. This can trigger neuroendocrine, immune, and metabolic reactions that cause oxidative stress and contribute to reduced meat quality [15]. High stocking density, like other types of stress, leads to activation of the hypothalamic–pituitary–adrenal (HPA) axis, resulting in increased corticosterone secretion [16,17]. This hormone can exert a dual effect on protein metabolism: it activates the ubiquitin–proteasome pathway (upregulating MuRF-1 and Atrogin-1/MAFbx), stimulating protein degradation, and suppresses the IGF 1/Akt/mTOR signaling pathway, inhibiting protein synthesis [17,18]. Furthermore, increased glucocorticoid production stimulates gluconeogenesis in the liver, utilizing amino acids—particularly alanine and glutamine—as substrates, thereby reducing their availability for deposition in muscle tissue [19]. In addition, high stocking density may restrict bird mobility, leading to mitochondrial dysfunction in skeletal muscles, impaired oxidative phosphorylation, and altered energy metabolism, which in turn affects amino acid utilization and protein turnover [20]. Therefore, understanding how stress modulates muscle physiology, influencing muscle tissue composition, is important for developing management strategies aimed at reducing stress and improving meat quality [21]. The available literature contains a limited number of studies examining the impact of poultry stocking density on the dynamics of amino acid content in broiler muscle tissue in relation to age.

While the present study focuses on amino acid composition, our parallel investigations using the same experimental model have revealed systemic biochemical alterations (hyperglycemia, increased bilirubin and triglycerides, decreased albumin) and tissue-level molecular changes (suppressed expression of Nrf2, GSH-Gpx, and HO-1) consistent with stress-related metabolic shifts [22,23,24]. These findings will be considered in the interpretation of the observed amino acid profile dynamics.

Dietary supplementation with bioactive compounds—adaptogens and antioxidants—represents a promising strategy for mitigating the negative impact of stressors of various etiologies on growth performance and meat quality. These compounds help enhance the body’s antioxidant defenses and reduce oxidative stress, thereby preserving the integrity of muscle structures and influencing the amino acid composition. For example, adding Cannabis sativa L. leaves to the diet of Ross-308 broilers has been shown to increase the content of free amino acids, including aspartic acid, serine, proline, methionine, and phenylalanine, which improved the palatability of the meat [25]. The effect of adding dried jujube fruit powder (DJFP) to the diet of Cobb broilers on the amino acid profile of muscle was studied. The level of essential amino acids was higher in the groups receiving this supplement (*p* < 0.05) [26].

Dihydroquercetin (DHQ) is a bioflavonoid found in small amounts in many plants. It was first isolated from larch wood in the 1960s by researchers in the former USSR [27]. Ascorbic acid, or vitamin C, is an essential vitamin with high antioxidant potential, which is due to its ability to inhibit lipid peroxidation in cell membranes and scavenge peroxyl radicals [28]. Vitamin E is the main antioxidant in the cellular antioxidant defense network, reducing the risk of lipid damage to cells and tissues caused by free radicals [29].

It is known that oxidative stress can affect the amino acid composition in the following ways. First, the side chains of amino acids are directly oxidized and undergo oxidative modification. Oxidized amino acids cannot be reused for protein synthesis. Secondly, the process of protein carbonylation makes them more susceptible to proteolytic degradation. Thirdly, lipid peroxidation products react with nucleophilic amino groups, reducing their bioavailability for protein synthesis. Fourth, oxidative stress depletes glutathione (GSH), the main intracellular antioxidant, increasing the metabolic demand for its precursor amino acids—cysteine, glutamate, and glycine [30]. Fifth, mitochondrial dysfunction induced by oxidative stress disrupts the tricarboxylic acid cycle, affecting the synthesis of interchangeable amino acids formed from intermediates [31]. Thus, oxidative stress creates conditions for changing the amino acid composition of muscle tissue through direct oxidation of specific residues, increased protein turnover, and competitive redistribution of amino acids for antioxidant protection, reducing their availability to structural proteins.

The use of adaptogen complexes is becoming increasingly important in poultry farming, as they provide a synergistic effect. Studying the biological properties of the developed adaptogen complex DHQEC (dihydroquercetin + vitamins E and C) is promising [32]. Research aimed at investigating the combined effects of DHQEC on the amino acid composition of poultry meat under simulated environmental conditions has not yet been conducted.

We hypothesized increased stocking density may alter the amino acid composition of muscle tissue in a muscle-type- and age-dependent manner. Dietary supplementation with the DHQEC complex (dihydroquercetin + vitamins E and C) is expected to mitigate these effects. We propose the efficacy of the adaptogens complex depends on the timing of its administration, with earlier supplementation (from day 1) providing greater protection than later supplementation (from day 21).

The aim of this study was to investigate the amino acid composition of muscle tissue (breast and thigh) of broiler chickens of the Russian cross Smena-9 under conditions of increased stocking density and with the use of a complex of adaptogens including dihydroquercetin, vitamins E and C. The research objectives also included studying the amino acid composition of muscle tissue in relation to age and sex.

The novelty of this study lies in the first comprehensive assessment of the total amino acid content in the breast and thigh muscles of Smena-9 broiler chickens under increased stocking density and dietary supplementation with the antioxidant complex DHQEC, with consideration of both age and sex effects.

## 2. Materials and Methods

### 2.1. Housing Conditions and Diet of Birds

The experiment was conducted on Smena-9 broiler chickens kept in the physiological yard of the Federal State Budgetary Scientific Institution “Federal Research Center for Animal Husbandry—All-Russian Research Institute of Animal Husbandry named after Academician L.K. Ernst” (Russia, Podolsk) in 2024. One hundred and twenty broiler chickens (50% cockerels and 50% pullets) were divided into four groups: S(-)CON, S(+)CON, S(+)DHQEC_21, S(+)DHQEC_1. The birds were kept in specialized BB-1 broiler cages (Stimul Group LLC, Pushkino, Russia).

The experimental design is presented in Table 1. A complete feed was used as the diet: starter feed for up to 11 days, grower feed from days 12 to 26, and finishing feed from days 27 to 52 (Table 2). The developed complex of dihydroquercetin + vitamins E and C (DHQEC) was added to the diet of S(+)DHQEC_21 and S(+)DHQEC_1 birds (from days 21 and 1 of the experiment). DHQEC is a powder containing DHQ (Ekostimul-2, Ametis JSC, Blagoveshchensk, Russia; DHQ content 72–73%) at a dose of 32 mg/kg of feed, vitamin E (INNOVIT E60, MEGAMIX Group, Russia) at 10 mg/kg of feed, and vitamin C (Tiger C 35, Anhui Tiger Biotech Co. Ltd., PVI-2-2.15/04504, Hangzhou, China) at 35 mg/kg of feed. A pilot batch of compound feed was prepared and mixed with DHQEC.

The stocking density was determined in accordance with the relevant standards for the Smena 9 meat cross [33] and was adjusted as the birds grew. On day 21, the stocking density in the S(-)CON group was 38 birds per m^2^; in the other groups, it was increased by 10%, reaching 42 birds per m^2^. Each week, the stocking density was adjusted as follows: 32 and 35 birds per m^2^ at 28–35 days, 21 and 23 birds per m^2^ at 35–42 days, and 17 and 19 birds per m^2^ from day 43 until the end of the experiment, respectively. The S(-) CON group was kept under the recommended stocking density for this cross (Stress-) (Table 3). For the S(+)CON, S(+)DHQEC_21, and S(+)DHQEC_1 groups, stocking density was increased by 10% from day 21 of age to create simulated environmental conditions. The cages used in our experiment measured 0.99 m in length and 0.61 m in width, providing a total area of 0.60 m^2^ per cage. The required cage area for all groups was adjusted using sliding plywood partitions as the birds grew. For each treatment, 30 birds were used for sampling and analyses (10 birds at each of the three slaughter ages: days 24, 34, and 52). At each sampling point, we randomly selected 5 males and 5 females per treatment to ensure balanced sex representation. The experimental unit for all statistical analyses was the individual bird, as birds were randomly sampled from the population and all measurements were performed on individual animals. Stocking density was adjusted weekly by moving temporary plywood partitions to maintain the target density as the birds grew. The number of birds per cage was as follows: during days 0–21, 23 birds per cage in the S(-)CON group (38 birds/m^2^) and 25 birds per cage in the S(+)CON group (42 birds/m^2^); during days 22–28, 19 birds per cage (32 birds/m^2^) and 21 birds per cage (35 birds/m^2^), respectively; during days 29–35, 19 and 21 birds per cage; during days 36–42, 13 and 14 birds per cage (21 and 23 birds/m^2^, respectively); and from day 43 to 52, 10 and 11 birds per cage (17 and 19 birds/m^2^, respectively). All cages within the same treatment group were maintained under identical environmental conditions (temperature, humidity, lighting, and ventilation) throughout the experiment.

Zoohygienic parameters met requirements (Table 4). To encourage water and feed consumption, lighting was provided for 23–24 h per day during the first 3–5 days. Thereafter, the light regime was 18–20 h and darkness was 4–6 h.

At 24, 34, and 52 days of age, 10 birds from each group were slaughtered. From day 24 onward, sex was determined visually prior to slaughter based on comb size and coloration, which are reliably distinguishable in broilers by this age. This allowed us to deliberately select 5 males and 5 females per treatment at each sampling point, ensuring a balanced subsample for sex-specific analyses. Following slaughter, the breast (pectoral) and thigh (femoral) muscles were collected from the right side of each carcass. Muscle tissue samples were cooled at +4 °C for 24 h and then homogenized. In the homogenized samples, dry matter content was determined according to GOST 33319-2015. Subsequently, the total amino acid composition of the muscles was determined in the dry matter.

### 2.2. Determination of the Amino Acid Composition of Muscle Tissue

The amino acid concentrations in the meat samples were determined using ion-exchange chromatography with post-column derivatization of samples with ninhydrin. A Prominence LC-20 high-performance liquid chromatography system (Shimadzu, Kyoto, Japan) was used, equipped with an ARM-1000 ninhydrin post-column derivatization reaction module (Sevko&Co, Moscow, Russia) and an ion-exchange resin column (Sevko&Co, Russia). Samples were prepared for analysis in accordance with GOST 32195-2013. Acid hydrolysis in 6 N HCl was used to decompose proteins into individual amino acids, with norleucine added as an internal standard. Hydrolysis was performed in fluoroplastic beakers with screw-on caps (CEM, Matthews, NC, USA) in a thermostat at 110 °C for 24 h. After hydrolysis, 160 µL of the resulting suspension was collected and evaporated at 110 °C to remove hydrochloric acid. Next, 1 mL of dilution buffer was added to the sample. The resulting suspension was centrifuged at 13.000 rpm for 5 min and then analyzed. To determine sulfur-containing amino acids (MET, CYS), the samples were treated with an oxidation mixture (formic acid with phenol as a preservative) before hydrolysis. The following amino acids were determined: aspartic acid (ASP), threonine (THR), serine (SER), glutamic acid (GLU), glycine (GLY), alanine (ALA), cysteine (CYS), valine (VAL), methionine (MET), isoleucine (ILE), leucine (LEU), tyrosine (TYR), phenylalanine (PHE), histidine (HIS), lysine (LYS), arginine (ARG), and proline (PRO). The values are expressed as grams of amino acids per 100 g of breast muscle.

The total amounts of essential amino acids (SEAA), nonessential amino acids (SNEAA), and flavor amino acids (SFAA; aspartic acid + glutamic acid + glycine + alanine + arginine) were calculated [10].

### 2.3. Statistical Processing

Data were analyzed statistically using Microsoft Office Excel 2003 and STATISTICA 10 (Statistica 13RU, StatSoft, Inc., Tulsa, OK, USA). Descriptive statistics, analysis of variance (ANOVA), and factor analysis were performed, followed by Tukey’s post hoc test for multiple comparisons. Regarding the statistical analysis: prior to ANOVA, normality of the data distribution was verified using the Shapiro–Wilk test, which is recommended for small sample sizes (n = 5). Homogeneity of variances was assessed using Levene’s test. Since all data met the assumptions of normality and homoscedasticity, one-way ANOVA was applied, followed by Tukey–Kramer post hoc tests for multiple comparisons. The Tukey–Kramer procedure was used to control the family-wise error rate (type I error) associated with multiple pairwise comparisons. To justify the adequacy of our sample size, we performed a post hoc power analysis based on the primary response variables. With n = 5 birds per sex × treatment × age combination, and using the observed means and pooled standard deviations, the achieved statistical power exceeded 80% at α = 0.05 for detecting the observed effect sizes. Mean values (M) and standard errors of the means (±SEM) were calculated. Differences were considered statistically significant at *p* < 0.05, and highly significant at *p* < 0.01 and *p* < 0.001. A factor ANOVA with main effects was conducted, and η^2^ (Partial Eta Squared) was calculated for the contribution of each factor (age, group, sex).

### 2.4. Animal Ethics Statement

The study was conducted in accordance with the principles of the European Convention for the Protection of Vertebrate Animals used for Experimental and other Scientific Purposes (ETS No. 123, Strasbourg, 1986). The research was approved by the bioethical commission of the L.K. Ernst Federal Research Center for Animal Husbandry (protocol № 3, dated 27 May 2022).

## 3. Results

### 3.1. Age-Related Changes in the Amino Acid Composition of Breast and Thigh Muscle

On day 24, glutamic acid was the most abundant amino acid in the breast muscle (12.34–13.04 g/100 g DM), whereas cysteine showed the lowest content (0.86–0.88 g/100 g DM; Table 5). The concentrations of Asp, Lys, and Leu ranged from 7.07 to 8.88 g/100 g, while arginine levels showed little variation across groups (6.21–6.44 g/100 g). The level of THR, VAL, and ILE varied from 4 to 5 g/100 g DM. The content of other amino acids in the breast averaged 2.5–3.5 g/100 g. The total amino acid content was lowest in S(+)DHQEC_21, which received an adaptogen supplement from day 21 of age. This group is also characterized by minimal contents of a number of both nonessential and essential amino acids: GLU, VAL, LEU, PRO. Moreover, for proline, the difference was significant relative to the S(-)CON group (*p* < 0.05) and S(+)CON (*p* < 0.05). A tendency toward a decrease in ASP and GLY concentrations in the experimental groups relative to the control was also observed (*p* = 0.06–0.07). In the S(+)DHQEC_1 group, which received an adaptogen supplement from the beginning of the experiment, the levels of most of the determined amino acids were slightly higher relative to the S(+)DHQEC_21 group. However, the overall amino acid profile of chicken breast in all groups changed insignificantly, suggesting the stability of its amino acid composition. The sum of flavor amino acids (ASP, GLU, GLY, ALA, and ARG) did not differ between the groups. The percentage of amino acids in the thigh muscle was similar to that in the breast muscle, but the quantitative composition differed, which was associated with a lower proportion of protein in the thigh. On the 24th day of the experiment, a marked decrease in the content of all amino acids in the experimental groups was observed compared to the control S(-)CON. In S(+)DHQEC_1 vs to S(-)CON, the content of THR (3.14 g/100 g versus 3.49 g/100 g) significantly (*p* < 0.05) decreased, ALA (4.53 g/100 g versus 5.06 g/100 g), CYS (0.71 g/100 g versus 0.79 g/100 g), MET (1.43 g/100 g versus 1.60 g/100 g), ILE (3.58 g/100 g versus 3.96 g/100 g), LEU (5.60 g/100 g versus 6.20 g/100 g), TYR (2.38 g/100 g versus 2.61 g/100 g) and HIS (1.87 g/100 g versus 2.04 g/100 g).

On day 34 of the experiment (Table 6), the total amino acid content in the breast and thigh of chickens was slightly higher than on day 24, which is associated with the development of muscle tissue during growth. In the breast, significant differences in the content of several amino acids were observed in the S(+)DHQEC_21 and S(+)DHQEC_1 groups relative to (-)CON and S(+)CON. Thus, the GLU level in S(+)DHQEC_21 and S(+)DHQEC_1 was significantly (*p* < 0.001) lower than in S(-)CON and S(+)CON. In the groups exposed to simulated housing conditions, the levels of GLY, MET, and PHE increased significantly relative to S(-)CON, with maximum values observed in S(+)DHQEC_1 (GLY: 3.88 vs. 3.32 g/100 g; MET: 2.38 vs. 1.90 g/100 g; PHE: 4.24 vs. 3.79 g/100 g; *p* < 0.001 for all). Also, in the S(+)DHQEC_21 and S(+)DHQEC_1 groups, a significant increase in the content of TYR and HIS was observed compared to the S(-)CON group. The amount of nonessential amino acids in the breast of poultry S(+)DHQEC_1 decreases compared to S(-)CON and S(+)CON (*p* < 0.001).

Similar dependencies in the amino acid content in the control and experimental groups of chickens were also observed in the thigh on day 34 of the study (Table 6). Thus, in the S(+)DHQEC_21 and S(+)DHQEC_1 groups, the level of ASP, THR, VAL, ILE, LYS significantly (*p* < 0.05) decreased, while the content of GLY, MET, TYR, PHE, HIS, ARG increased, as well as Pro for the S(+)DHQEC_21 group (3.18 g/100 g versus 2.55 g/100 g in the control, *p* = 0.0018). The SAA in the thigh from experimental groups increased significantly by day 34 and approached the values in the control group (73.77 g/100 g—S(-)CON, 71.30 g/100 g—S(+)CON, 71.57 g/100 g—S(+)DHQEC_21, 71.14 g/100 g—S(+)DHQEC_1), which differs significantly from the trends in the thigh on day 24 (Table 5).

The breast muscle of broiler chickens exposed to stocking-density stress (Table 7) showed a decrease in SER content (*p* = 0.009), GLY, ALA content decreased compared to S(-)CON and S(+)DHQEC_1 in the S(+)CON and S(+)DHQEC_21 (*p*=0.01). MET (*p* < 0.001), ILE (*p*=0.016), TYR (*p* < 0.001) and HIS (*p* = 0.026) content in the groups exposed to stress was significantly higher at the age of 52 days. The most pronounced changes upward were noted in the poultry that received the adaptogens complex from the 1 st day of the experiment (S(+)DHQEC_1). In the S(+)DHQEC_1 group, an increase in the sum of essential amino acids in the breast muscle was noted (*p* = 0.04). Less pronounced differences between the groups were observed in the thigh meat at 52 days of age of the birds (Table 7). The content of GLY and ALA in the S(+)DHQEC_1 group of birds was higher compared to S(+)CON (for GLY at *p* < 0.05 and for ALA at *p* < 0.01) and S(+)DHQEC_21 (for GLY at *p* < 0.001 and for ALA at *p* < 0.05).

The influence of the “age” factor had an uneven effect on the amino acid composition of poultry meat in different groups (Table 8). In breast muscle in the S(-)CON group, most of the reliable age-related differences are observed in ASP (*p* < 0.001), SER (*p* < 0.001), GLU (*p* < 0.001), GLY (*p* < 0.001), ALA (*p* = 0.008), CYS (*p* = 0.027), VAL (*p* < 0.001), MET (*p* < 0.001), ILE (*p* < 0.001), TYR (*p* < 0.001), PHE (*p* < 0.001), HIS (*p* = 0.019), ARG (*p* = 0.016), PRO (*p* = 0.02) content. In the breast of the birds of the S(+)CON group, age had a significant effect on the content of ASP (*p* < 0.001), THR (*p* = 0.01), GLU (*p* < 0.001), GLY (*p* < 0.001), ALA (*p* < 0.001), CYS (*p* = 0.004), VAL (*p* = 0.002), MET (*p* < 0.001), ILE (*p* < 0.001), TYR (*p* < 0.001), PHE (*p* < 0.001), HIS (*p* = 0.019), PRO (*p* < 0.001). In the breast of birds exposed to stress and receiving adaptogens from the 21st day of age, age-related changes affected the content of the following amino acids: GLU (*p* = 0.00081), GLY (*p* < 0.001), MET (*p* < 0.001), LEU (*p* = 0.004), TYR (*p* < 0.001), PHE (*p* = 0.004), HIS (*p* < 0.001), PRO (*p* < 0.001). In the S(+)DHQEC_1 group, there were more significant differences with age in the breast muscle: GLY (*p* < 0.001), ALA (*p* = 0.020), VAL (*p* = 0.002), MET (*p* < 0.001), ILE (*p* = 0.006), TYR (*p* < 0.001), PHE (*p* < 0.001), HIS (*p* < 0.001), ARG (*p* < 0.001). Age significantly affected the total amount of amino acids and nonessential AA in all poultry groups.

A different pattern was observed in the thigh muscle. In the negative and positive control groups (S(-)CON and S(+)CON), changes with age concern only the following amino acids: GLY (*p* < 0.001 and *p* = 0.007), MET (*p* < 0.001), TYR (*p* = 0.004 and *p* < 0.001), PHE (*p* = 0.004 and *p* = 0.005), HIS (*p* < 0.001). More significant changes in thigh amino acid were observed in the groups receiving adaptogens. The sum of essential amino acids did not change significantly with age. The sum of flavor AA significantly changed with age in the breast of poultry of all groups, except for S(+)DHQEC_1. The total sum of amino acids, as well as essential and nonessential ones, significantly changed with age in the S(+)DHQEC_21 and S(+)DHQEC_1 groups, and the sum of flavors is only in the S(+)DHQEC_1 group.

### 3.2. The Effect of Sex on the Amino Acid Composition of Muscle Tissue

Analysis of the data in Table 9 shows that a significant difference in the amino acid content in the breast muscle depending on the bird’s sex is observed only at 24 days of age and for GLY at 52 days of age (*p* = 0.03). This table presents the combined data for 4 groups (n = 40). At 24 days, a significant difference is observed for THR (*p* = 0.03), GLU (*p* = 0.03), ALA (*p* = 0.02), VAL (*p* = 0.03), ILE (*p* = 0.02), LEU (*p* = 0.02), PHE (*p* = 0.02), and HIS (*p* = 0.03). Between cockerels and pullets at this age, there were also significant changes in the total amino acid content (*p* = 0.03), the total amount of nonessential amino acids (*p* = 0.04), essential amino acids (*p* = 0.02), and flavor amino acids (*p* = 0.02). In the thigh muscle, the main significant changes by sex were observed at the age of 52 days and concerned the content of GLY (*p* = 0.03), LEU (*p* = 0.04), HIS (*p* = 0.03), LYS (*p* = 0.004), PRO (*p* = 0.01), total amino acids (*p* = 0.03), total nonessential amino acids (*p* = 0.02), essential (*p* = 0.03), and flavor (*p* = 0.04). At the age of 34 days, only the content of GLU changed significantly by sex (*p* = 0.04). Thus, the effect of sex on the amino acid composition of muscle tissue was less pronounced than age and alimentary factors. The fact that the amino acid composition of the breast muscle at the age of 24 days is subject to sex-related changes indicates unequal nitrogen metabolism in cockerels and pullets.

### 3.3. Tissue Effect

Both the content of individual amino acids and their totals depended significantly on the muscle tissue type—breast or thigh (Table 10). The exceptions are the content of PRO at 24 days of age and GLY at 52 days of age. Breast meat has a higher amino acid content.

### 3.4. Relative Contribution of Each Factor

**Figure 1:**
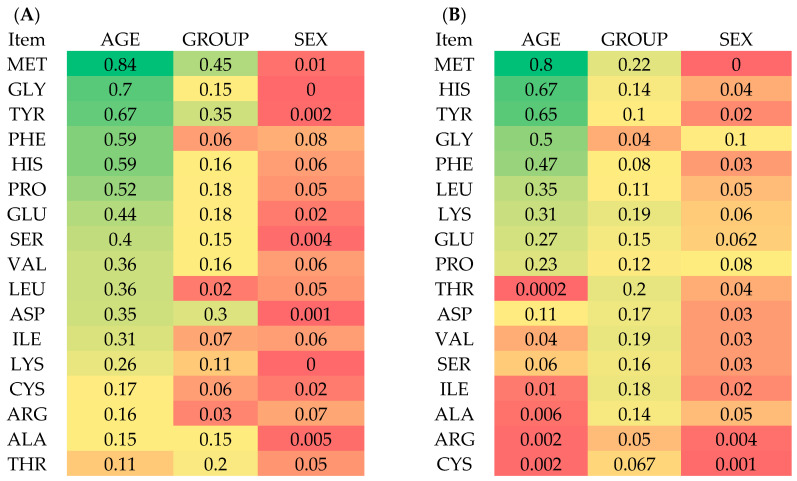
Heatmap of η^2^ values from three-way ANOVA showing the effects of age, group, and sex on amino acid content in the breast (**A**) and thigh (**B**) of broiler chickens. The relative contribution of each factor is shown.

Next, we conducted a three-way ANOVA to determine the contribution of each factor (age, group, and sex) to the amino acid composition of breast and thigh meat (Figure 1). Age is the most significant factor for the amino acid composition of breast meat. The highest η^2^ values were observed for MET (0.84), GLY (0.70), TYR (0.67), PHE and HIS (0.59), and PRO (0.52). Group also significantly affected the breast meat content of MET (0.45), ASP (0.30), TYR (0.35), THR (0.20), PRO (0.18), VAL (0.16), HIS (0.16), SER (0.15), and LYS (0.11). Sex significantly affected the content of the following amino acids: VAL (η^2^ = 0.06 at *p* = 0.04). ILE (η^2^ = 0.06 at *p* = 0.03). PHE (η^2^ = 0.08 at *p* = 0.02). HIS (η^2^ = 0.06 at *p* = 0.04). ARG (η^2^ = 0.07 at *p* = 0.02). PRO (η^2^ = 0.05 at *p* = 0.05). As in breast meat, in thigh meat, age is the dominant factor influencing the amino acid composition, but its influence is less pronounced. Age significantly affected (*p* < 0.001) the content of GLU (0.27), GLY (0.50), MET (0.80), LEU (0.35), TYR (0.65), PHE (0.47), HIS (0.67), LYS (0.31) and PRO (0.23) in thigh meat. The group is in second place and is significant for amino acids content: ASP (0.17 at *p* = 0.002), THR (0.20 at *p* = 0.001), ALA (0.14 at *p* = 0.08), VAL (0.19 at *p* = 0.001), MET (0.22 at *p* < 0.001), ILE (0.18 at *p* = 0.001), LEU (0.11 at *p* = 0.03), TYR (0.10 at *p* = 0.04), HIS (0.14 at *p* = 0.01), LYS (0.19 at *p* < 0.001), PRO (0.08 at *p* = 0.01). The relative contribution of the “group” factor is within 0.1–0.22. A significant effect of age and group was observed for the following amino acid levels: ASP, GLU, MET, LEU, TYR, HIS, LYS, and PRO. Sex significantly influenced the following amino acid levels in the thigh muscle: GLU (0.062), GLY (0.10), ALA (0.05), LYS (0.06), and PRO (0.08). All differences were significant at *p* < 0.05. The relative contribution of the “sex” factor was up to 0.1.

## 4. Discussion

The amino acid composition is an indicator of the nutritional value of meat. It is influenced by age, housing conditions, and bird diet. In this study, we evaluated the effects of stocking density and adaptogen supplementation at three developmental stages to capture the temporal dynamics of these effects and to ensure that our findings are placed in their proper biological context. The results of this study demonstrate that the simulated environmental conditions created by a high stocking density had differential effects on the amino acid composition of the different muscle types (breast and thigh). The most pronounced changes were observed in the thigh muscle at the very beginning of the exposure, at 24 days of age. The use of the developed adaptogen complex, which includes dihydroquercetin, vitamins E and C—especially when fed from an earlier age—mitigated the impact of increased stocking density conditions and contributed to maintaining the levels of flavor-related and essential amino acids by the end of the rearing period of Smena-9 broiler chickens.

Broiler chickens are considered more susceptible to adverse environmental conditions, likely due to their higher metabolic rate associated with rapid growth [34]. A comparison of the S(–)CON and S(+)CON groups indicated that the housing conditions with increased stocking density, which are generally considered simulated in the literature [14,35], affected the amino acid composition of the breast and thigh of broiler chickens in our study manifested itself at 34 days of age (2 weeks after the start of exposure). At this age, the negative change in environmental conditions led to an increase in TYR in the breast meat of birds in the S(+)CON group compared to S(–)CON (*p* < 0.05, PRO (*p* < 0.05), and in thigh meat—an increase in LYS (*p* < 0.05).

When meat samples were collected at 52 days of age, we observed no differences between the S(+)CON and S(–)CON groups. This may indicate an adaptive response of the birds to the simulated housing conditions.

In the thigh muscle on day 24 of the experiment, a pronounced decrease in the content of all amino acids was observed in the groups exposed to simulated environmental conditions and receiving the adaptogen complex from day 1 of the experiment, S(+)DHQEC_1, compared to S(–)CON. This suggests that the adaptogens had a marked effect on the amino acid profile of the thigh muscle in broilers under the simulated conditions of a high stocking density during the period of intensive growth. No such changes were observed in the breast muscle. This indicates a greater susceptibility of the thigh muscle to environmental conditions and alimentary factors. This trend may be due to several factors.

In general, the mechanisms described in the literature for heat stress are similar for any chronic stress (including crowding) [35], since they are mediated by the same hormones—glucocorticoids [16]. Corticosterone directly activates signaling pathways responsible for muscle protein breakdown (e.g., the ubiquitin–proteasome pathway). Studies in broilers have shown that stress increases the expression of the muscle atrophy F-box gene (a marker of muscle atrophy). High levels of corticosterone also suppress the IGF-1/Akt/mTOR signaling pathway, leading to reduced synthesis of new amino acids in muscle tissue. Thus, chronic overcrowding stress not only accelerates protein breakdown but also reduces the rate of new protein synthesis [17]. It should be noted, however, that our parallel study did not reveal statistically significant changes in serum corticosterone levels in the same birds [22]; therefore, the involvement of systemic glucocorticoid signaling in the observed amino acid changes remains speculative. More convincingly, our independent molecular data demonstrated significant downregulation of antioxidant defense genes (*Nrf2, GSH-Gpx, HO-1*) in intestinal and liver tissues under high stocking density [24], suggesting local oxidative stress at the tissue level, which may contribute to altered amino acid metabolism. Fei Li et al. (2024) reported that chronic stress damages the intestinal barrier, alters the microbiota composition (fewer beneficial Firmicutes, more Proteobacteria), and impairs nutrient absorption, including amino acids from feed [18]. Moreover, the high energy expenditure of birds to adapt to simulated conditions inevitably leads to an energy deficit in the body. To compensate for this, gluconeogenesis is activated in the liver to synthesize glucose from non-carbohydrate precursors, which is consistent with the findings of Ma B. et al. (2021) [17].

In addition, increased stocking density may contribute to reduced locomotor activity of birds and, consequently, affect the metabolic status of the limb muscles, including the thigh. It is known that with reduced locomotor activity, changes in energy metabolism may be observed in the thigh muscle of broiler chickens, manifested as impaired mitochondrial function and oxidative phosphorylation [19]. This leads to decreased energy production. Furthermore, a reduction in antioxidant levels in muscles may occur, which promotes the development of oxidative stress [19]. Reduced locomotor activity may lead to the appearance of oxidative-type fibers as an adaptive response to hypoxic conditions [20]. This is especially pronounced in modern poultry crosses, since selection for rapid growth leads to a decrease in the adaptive capacity of the birds.

The decrease in the methionine level—a key amino acid with antioxidant action that is a precursor of glutathione—in the thigh muscle of birds exposed to simulated high-stocking-density conditions is consistent with the hypothesis of increase in oxidative stress levels in this muscle. In the study by El-Tarabany M.S. et al. under chronic heat stress, the levels of all essential amino acids in the breast of Ross broiler chickens were significantly reduced, with the exception of threonine, tyrosine, and phenylalanine [36]. The authors attribute this to elevated corticosterone levels in heat-stressed birds, which suppresses protein synthesis and accelerates its breakdown. In addition, heat stress accelerates the utilization of amino acids as metabolic fuel to provide energy through gluconeogenesis in the liver. It has been observed that heat stress increases the methionine requirement of broilers [37]. The use of dietary methionine in poultry diets under increased stocking density helps reduce oxidative stress in the birds [30].

In other studies, rearing conditions also influence the amino acid composition of broiler meat. For example, under floor housing, on day 49, a decrease in lysine content and an increase in arginine content are observed in both white and red meat [38]. The level of glutamate (Glu), an amino acid that plays a decisive role in flavor formation, was significantly higher in the free-range chicken group than in the cage-housed group (*p* < 0.05) [13].

Adaptogens have recently found wide application in animal husbandry practice [39,40], and their use in poultry farming has begun recently [41,42]. The adaptogenic properties of the components of DHQEC (dihydroquercetin, vitamins E and C) have already been described previously [43,44,45,46]. In our previous studies on monogastric animals, dietary supplementation with the complex at 0.025% of the basal diet throughout the entire rearing period improved immune responsiveness and stress resistance in pigs, resulting in enhanced growth performance [47]. An improvement in the microstructure of pig muscle tissue through the use of DHQEC in vivo has been demonstrated, suggesting a positive effect of the adaptogen complex on animal stress resistance and the degree of glycolysis in meat [48].

We have previously described the effects of simulated housing conditions on the antioxidant and hormonal status of broiler chickens [22]. In the present study, we evaluated the effect of DHQEC on the amino acid profile of the breast and thigh muscles of broilers under simulated environmental conditions during ontogeny.

The use of DHQEC in broiler diets, both from 21 days of age and from day 1 of the experiment, contributed to a decrease in PRO content in the breast of birds at 24 days of age. In the S(+)DHQEC_21 group, the content of this amino acid decreased compared to S(–)CON (*p* < 0.05) and S(+)CON (*p* < 0.05). In the S(+)DHQEC_1 group, the PRO content tended to decrease compared with the S(–)CON and S(+)CON groups.

At 34 days of age, when adaptogens were fed both from day 1 of the experiment and from 21 days of age, the level of GLU in the breast muscle decreased compared to the S(–)CON group (*p* < 0.001 and *p* < 0.05, respectively). The levels of GLY and TYR, on the contrary, increased in the groups of birds kept under simulated environmental conditions (S(+)CON) and receiving the adaptogen complex (groups S(+)DHQEC_21 and S(+)DHQEC_1).

At 52 days of age, in the breast, the level of MET increased under simulated environmental conditions, and increased even more with the use of adaptogens. In the thigh, it did not change.

The improvement in the amino acid profile, resulting from both an increase in the total amino acid content and individual functional amino acids such as methionine, phenylalanine, histidine and arginine, may be attributed to the birds’ adaptation to stress conditions and the positive effect of the adaptogen complex supplementation.

The increase in the methionine levels in the breast and thigh muscles of the birds receiving the adaptogen complex indicated a reduction in oxidative stress levels. Based on the results of previous studies on the use of the DHQEC complex in the nutrition of monogastric animals and poultry, and the demonstrated positive effects on the antioxidant status of the birds [22,47], it can be assumed that the impact of environmental conditions is mitigated through the action of the DHQ components and vitamins E and C. This may lead to a reduced requirement for methionine—which plays a key role in the antioxidant defense system—for these purposes, potentially contributing to its increased content in muscle tissue. However, direct evidence linking these changes to decreased oxidative stress in muscle tissue was not obtained in the present study and requires further investigation.

The trend toward an increase in histidine concentration in the muscle tissue of broiler chickens receiving the DHQEC complex also indicates an adaptive response of the birds aimed at enhancing antioxidant properties under the action of adaptogens. The role of histidine as a precursor for carnosine synthesis is known [49].

The concentration of free and protein-bound amino acids in muscle tissue reflects a dynamic equilibrium between exogenous supply, endogenous synthesis, and catabolic losses. Our previous findings indicate that dietary adaptogens improved antioxidant defense mechanisms, thereby mitigating oxidative damage to muscle proteins and sparing sulfur-containing amino acids (particularly methionine and cysteine) from excessive utilization in glutathione biosynthesis. At the same time, enhanced immune competence and improved proteolytic digestibility of the diet [22] promoted a greater postprandial amino acid flux into the portal circulation. Consequently, the net change in individual amino acid concentrations in muscle tissue can be attributed to the synergistic action of augmented intestinal uptake and diminished oxidative degradation, rather than to dietary intake alone.

One manifestation of the adaptive response in broilers to simulated environmental conditions was an increase in the glycine levels in the breast and thigh muscles at 34 days of age and in the breast muscle at 52 days of age. Glycine is an important component for collagen synthesis [50]. The effect of increased stocking density may lead to enhanced breakdown of protein structures, while adaptogens stimulate fibroblasts to produce collagen, which requires increased levels of its constituent—glycine.

In another study under heat stress conditions, a high dose of an adaptogen (1 kg/1000 kg of diet) based on a combination of the herbs *Ocimum sanctum*, *Withania somnifera*, and *Emblica officinalis* significantly reduced the levels of glutamic acid, glycine, serine, and threonine in the breast muscle of Cobb 500 broiler chickens compared to birds subjected to this type of stress and receiving a control diet.

With age, the levels of all protein amino acids increased in both the breast and thigh meat of the birds (Table 5 and Table 7). The total amino acid content in chicken breast increases at 34 days of age compared to 24 days of age (from 82.36–85.39 to 86.29–89.94 g/100 g). By day 52, this value remains at that level or decreases slightly. In the work of Bychaev A.G. (2019) [51], it is also reported that the meat of chickens slaughtered at earlier ages contains fewer amino acids, especially essential ones. Analysis of broiler meat from birds slaughtered at an early age showed that in terms of total essential amino acids (g/100 g), meat from 45-day-old chickens has a clear advantage over meat from 35-day-old chickens: +0.230 and +0.094 versus +0.600 and +0.958 for cockerels and pullets in breast meat and drumsticks, respectively [51].

In our study, the sum of flavor-related amino acids (flavor amino acids = aspartic acid + glutamic acid + glycine + alanine + arginine) was calculated [10]. The involvement of these amino acids in flavor formation has been described by various researchers [52,53,54,55,56].

In the S(+)DHQEC_1 group, the total amino acid content at 34 days was 88.71 g/100 g, which is higher than in the previous age period at 34 days (86.26 g/100 g). In the breast of all bird groups except S(+)DHQEC_1, the level of flavor amino acids increased by day 34 of age and then decreased to the level of 24 days of age by day 52. This indicates that with age, poultry meat changes its composition toward a decrease in amino acid content, and the optimal period for slaughter of birds of this cross may be between 34 and 50 days of age, which would allow obtaining more complete meat raw material from these birds. In the group of birds receiving adaptogens from day 1 of the experiment, the level of flavor amino acids at 52 days remained at the level of 34 days and did not decrease, indicating stabilization of the amino acid composition of muscle tissue under the action of the adaptogen complex and preservation of the flavor properties of the raw material with age.

The total amino acid content in the thigh showed the same trend of increase by day 34 compared to day 24 in all bird groups. By day 52, the increase in total amino acid content continued. In the group of birds exposed to simulated high-stocking density conditions, the total amino acid content approached the values of the S(–)CON group, which may indicate adaptation of the birds to stress conditions and the action of adaptogens.

With age, the levels of MET, TYR, PHE, and HIS increase in both the breast and thigh muscles of birds. This is especially noticeable in the S(+)CON, S(+)DHQEC_21, and S(+)DHQEC_1 groups.

The increase in meat amino acid profile parameters with age is due to several factors. This may be associated with changes in the proportions of amino acid requirements for maintenance and growth in chickens. During meat maturation (autolysis), free amino acids (glutamic acid, arginine, threonine, phenylalanine, etc.) accumulate in the meat. Also, with age, the optimal ratio between amino acids changes.

In the study by Li J. et al. (2022), histidine and isoleucine showed an increasing trend with age, while aspartic acid decreased with age [57].

In contrast to our results, the study by Suliman G.M. et al. (2023) showed that amino acid content was higher in 35-day-old birds than in 49-day-old ROSS-308 broiler chickens [58].

In our study, regardless of muscle type, the amino acids that changed most with age in all groups were methionine, glycine, tyrosine, phenylalanine, and histidine.

The breast meat was characterized by a higher amount of amino acids in all age periods. The total amino acid content in the breast was 82.36–85.09, 86.29–89.94, and 86.94–88.71 g/100 g at 24, 34, and 52 days, respectively, while in the thigh these values were 65.76–72.665, 71.14–73.77, and 73.10–77.80 g/100 g, respectively. The total content of flavor amino acids was also higher in the breast, which highlights the higher flavor qualities of this muscle group in our study. Other studies have also compared the amount of flavor amino acids responsible for umami in different parts of chicken carcasses [59]. Meanwhile, in Guangyuan Grey chickens, on the contrary, the umami amino acid content was higher in the thigh, especially in 120-day-old cockerels [8].

Regarding essential amino acids, almost all of them were higher in the breast muscle. The exception was methionine content in some bird groups at 34 and 52 days of age. The most noticeable differences between muscle groups were observed for leucine, lysine, and valine.

The thigh meat was less subject to changes in amino acid composition. This suggests that with age, the amino acid composition becomes more stable.

The differences in amino acid composition are likely due to the structural characteristics and functions of the breast and thigh muscles. Muscle fibers in the breast are thicker and rich in contractile proteins required for short “burst” efforts (e.g., wing flapping), while connective tissue is less abundant. The thigh, in turn, contains many “slow” fibers and connective tissue. The thigh is constantly working when the bird walks and stands, so it contains more collagen and elastin. This explains the high levels of glycine, threonine, and serine in the thigh, which participate in the synthesis of connective tissue proteins [60]. In addition, the biceps femoris muscle contains more intramuscular fat than the superficial breast muscle, which is explained by the increased functional load on the former muscle, which requires more energy [61,62].

In the study by Yin L. et al. (2023), it was shown that the different amino acid contents in the thigh and breast of Guangyuan grey chickens provide different nutritional values and flavor qualities [8]. The authors demonstrated that the thigh muscle had the highest glutamine content compared to the breast, in which histidine content was the highest. Amino acids that impart umami and sweet flavors to meat predominated in the thigh. The essential amino acid content was higher in the breast muscle.

In the study by Ou Z. et al. (2022), the essential amino acid content was also higher in the breast muscle, while the total flavor amino acid content was higher in the thigh of Wuliangshan Black-bone chickens compared to the breast [10].

Breast meat contains more essential and fewer nonessential amino acids compared to thigh meat, which is consistent with our data and the results of other researchers [61,62].

In the study by Liu X. et al. (2026), 28-week-old male Dong’an chickens showed higher amino acid (GLY, ALA, PRO) and PUFA contents in the thigh muscles, as well as lower WLR, crude fat, and MUFA contents in the breast muscles [63].

Numerous studies confirm the differences in amino acid composition between the breast and thigh [62,64], which is fully consistent with our results. It can be assumed that the differences in the amino acid composition of muscle tissue are due to the anatomical, morphological, and functional characteristics of the breast and thigh muscles. The thigh is rich in glycine, hydroxyproline, hydroxylysine, threonine, serine, as well as aspartic acid, glutamic acid, alanine, cysteine, and tyrosine. However, the breast contains larger amounts of several functional and essential amino acids: valine, leucine, isoleucine, histidine, lysine, methionine, threonine, tryptophan, and phenylalanine. In particular, it has been reported that the yield of breast muscle is significantly influenced by lysine and methionine, which explains their greater accumulation in the breast [65].

For the breast muscle, age was the main factor influencing the amino acid composition. Thus, a significant difference (*p* < 0.05) was obtained for 12 of the 17 determined protein amino acids: ASP, SER, GLU, GLY, MET, LEU, TYR, PHE, HIS, LYS, ARG, PRO. FOR ALA, CYS, VAL, and ILE, pronounced trends close to significant were revealed depending on age. THR was the only amino acid for which no significant relationship with age was found. The factor “group” was statistically significant for 7 of the 17 amino acids: ASP, THR, GLU, ALA, VAL, ILE, LYS. FOR SER, MET, and PRO, a pronounced trend close to significance was observed. Dependence of amino acid composition on sex was observed only for threonine (F = 4.597, *p* = 0.036). It can be concluded that the amino acid profile of the breast depends to a greater extent on the age of the birds.

Somewhat different trends were observed in the thigh. The factor “age” had a significant effect on 8 of the 17 amino acids: GLU, MET, LEU, TYR, PHE, HIS, LYS, and PRO. For 11 amino acids, a relationship with the factor “group” was found: ASP, THR, SER, GLU, ALA, VAL, ILE, LEU, LYS, ARG, and PRO. No dependence of amino acid content in the thigh on sex was found. Thus, the presence of the stress factor had a greater influence on the amino acid composition of the thigh muscle. In the thigh muscle, the effect of age on amino acid composition was also less pronounced.

Age and group had significant effects on the content of the following amino acids in the breast: ASP (*p* < 0.001), THR (*p* = 0.02 and *p* = 0.001), SER (*p* = 0.006 and *p* = 0.008), GLY (*p* < 0.001 and *p* = 0.008), ALA (*p* = 0.003 and *p* = 0.009), VAL (*p* < 0.001 and *p* = 0.005), MET (*p* < 0.001), TYR (*p* < 0.001), HIS (*p* < 0.001 and *p* = 0.006), LYS (*p* < 0.001 and *p* = 0.04), PRO (*p* < 0.001 and *p* = 0.003).

Thus, age is the dominant factor influencing the amino acid composition of both the breast and the thigh. The use of adaptogens and simulated environmental conditions had a moderate effect. The sex factor had the least pronounced effect on the amino acid composition of muscle tissue compared to age and housing and feeding conditions. The effect of sex was mainly manifested at an early age (24 days) and in the breast muscle. This indicates different levels of metabolic changes during the maturation of cockerels and pullets. Similar results were obtained by other authors [58]. These findings have important practical implications and indicate that separating carcasses by sex is unnecessary.

Finally, beyond the mechanistic interpretation, the practical relevance of the observed amino acid changes deserves explicit consideration. Although the absolute differences between groups were numerically modest in some cases, their nutritional and commercial impact is substantial when evaluated in the context of specific amino acid functions. The most robust finding was the increase in methionine content—the first limiting amino acid in poultry meat—in the breast muscle at 52 days. In the S(+)DHQEC_1 group, methionine increased by 16.6% (from 2.17 to 2.53 g/100 g dry matter, *p* < 0.001) compared with the recommended-density control group (S(–)CON). This increment is nutritionally meaningful because methionine directly influences protein quality scores (e.g., PDCAAS) and is critical for glutathione biosynthesis, which plays a key role in antioxidant defense. From a practical standpoint, a 16.6% increase in the methionine content of meat reduces the need for synthetic methionine supplementation in feed formulations—a direct economic benefit, given that methionine is one of the most expensive feed additives. Even more pronounced effects were observed in the thigh muscle at 34 days, where methionine increased by 27.6% (from 1.63 to 2.08 g/100 g, *p* < 0.001) in the S(+)DHQEC_1 group compared with S(–)CON. This indicates that the adaptogen complex is particularly effective in protecting thigh muscle protein from stress-induced catabolism during the early stages of exposure, which is commercially relevant because thigh meat constitutes a substantial proportion of the carcass value. Regarding essential amino acid balance, the sum of essential amino acids (SEAA) in breast meat at 34 days increased by 2.2% (from 42.54 to 43.48 g/100 g, *p* < 0.001) in the high-density control group (S(+)CON) compared with S(–)CON. Although this increase is modest, it reflects a physiological adaptation to stress that may involve the mobilization of amino acids from other tissues. Notably, this effect was not observed in the adaptogen-supplemented groups, suggesting that adaptogens may redirect amino acids toward other metabolic needs, such as antioxidant defense or immune function. In contrast, high stocking density had a detrimental effect on lysine content in the thigh muscle at 34 days, with a 9.1% decrease (from 7.39 to 6.72 g/100 g, *p* < 0.05) in the S(+)CON group compared with S(–)CON. This depletion of lysine—the second limiting amino acid—is nutritionally concerning, as lysine is essential for growth and tissue repair. The adaptogen complex partially prevented this loss, although the effect was not statistically significant, suggesting that later administration may be less effective in protecting lysine reserves. Finally, while no statistically significant differences were observed for flavor-related amino acids (SFAA), we noted a biologically relevant trend: SFAA content in the S(+)DHQEC_1 group was maintained between days 34 and 52, whereas it declined in the other groups. This stabilization suggests that early adaptogen administration may help preserve sensory quality during extended rearing periods—a commercially valuable benefit, as it allows producers to achieve higher carcass weights without compromising meat palatability. Collectively, these findings indicate that although the absolute changes in individual amino acids are often modest, their cumulative effect on nutritional quality, feed efficiency, and product value is both tangible and commercially meaningful. The early administration of the DHQEC adaptogen complex appears to be a practical strategy for maintaining meat quality under intensive production conditions, particularly by protecting methionine reserves and stabilizing flavor-related amino acid profiles.

## 5. Conclusions

The results of this study demonstrate that the amino acid composition of the breast and thigh muscles of Smena 9 broiler chickens changed significantly depending on the slaughter age, stocking density, and the use of the adaptogen complex. In the breast muscle, a higher total content of amino acids, essential amino acids, and flavor amino acids was noted in all studied samples compared to the thigh. At the same time, the thigh muscle is less susceptible to age-related changes. With age, the total amino acid content in the poultry meat increased, with methionine, glycine, tyrosine, phenylalanine, and histidine showing the most noticeable changes. The adaptogen complex (DHQEC) had a positive effect on the amino acid composition of the meat, especially when administered from day 1 of the experiment. This was manifested in the stabilization of flavor amino acid content by 52 days of bird life, an increase in the total essential amino acids by this age, and an increase in the content of methionine, histidine, and glycine in the thigh, which enhances biological value. The use of the complex from 21 days of age had a less pronounced effect on the amino acid composition but had a positive effect on the content of sulfur-containing amino acids. Thus, to obtain meat from Smena 9 broilers with a high content of essential and flavor amino acids, it is recommended to slaughter birds at 34–52 days of age, regardless of sex. Early administration of the adaptogen complex (comprising dihydroquercetin and vitamins E and C) can mitigate the negative impact of simulated housing conditions and help preserve a rich amino acid composition. These findings may contribute to the development of broiler rearing practices aimed at improving the amino acid composition of meat and may inform decisions on optimal slaughter age and sex-related effects. We acknowledge that the mechanistic interpretations proposed in this study are based on established physiological pathways and are supported by our parallel biochemical and molecular data. However, direct measurements of systemic stress hormones, circulating reactive oxygen species, or muscle protein degradation markers were not performed in the present work. Therefore, the specific mechanisms linking the observed amino acid changes to systemic stress responses remain hypothetical. Further targeted studies are needed to directly quantify glucocorticoid receptor signaling, oxidative stress markers in muscle tissue, and protein turnover rates under crowding stress.

## Figures and Tables

**Table 1:** Experimental design.

Group	Age	Feeding Ration	Housing Condition
Phase 1			
S(-)CON	0–11 days	Starting compound	No high-stocking-density factor
S(+)CON	0–11 days	Starting compound	No high-stocking-density factor
S(+)DHQEC_21	0–11 days	Starting compound	No high-stocking-density factor
S(+)DHQEC_1	0–11 days	Starting compound + DHQEC	No high-stocking-density factor
Phase 1			
S(-)CON	12–26 days	Growth	No high-stocking-density factor
S(+)CON	12–26 days	Growth	Stocking density increased by 10% from day 21
S(+)DHQEC_21	12–26 days	Growth + DHQEC from day 21	Stocking density increased by 10% from day 21
S(+)DHQEC_1	12–26 days	Growth + DHQEC	Stocking density increased by 10% from day 21
Phase 3			
S(-)CON	27–52 days	Finishing	No high-stocking-density factor
S(+)CON	27–52 days	Finishing	Stocking density increased by 10%
S(+)DHQEC_21	27–52 days	Finishing + DHQEC	Stocking density increased by 10%
S(+)DHQEC_1	27–52 days	Finishing + DHQEC	Stocking density increased by 10%

**Table 2:** Composition of the basic diet for broilers.

Item	Unit	Starting Compound	Growth	Finishing
Wheat	%	35.00	40	55
Corn (Maize)	%	24.00	20	10
Wheat bran	%	-	-	5
Full-fat soybean	%	-	9	5
Soybean meal (SBM)	%	26.00	9	10
Sunflower meal (sunflower cake)	%	5.00	14.4	10
Sunflower oil	%	0.60	-	-
Fishmeal	%	1.70	-	-
Feed yeast	%	3.00	3	3
L-Lysine monohydrochloride 98%	%	0.20	0.2	0.2
DL-Methionine 98%	%	0.30	0.3	0.3
Monocalcium phosphate (MCP)	%	1.20	1.2	1.2
Limestone (feed-grade calcium carbonate)	%	1.80	1.8	1.8
Salt (sodium chloride)	%	0.20	0.2	0.2
Premix P5-1	%	1.00	1	1
Nutritional values				
Metabolizable Energy	Kcal/100 g	308.00	313.00	315.30
Metabolizable Energy	MJ/kg	12.90	13.10	13.20
Dry Matter	%	89.42	89.28	91.47
Crude Protein	%	22.91	21.03	19.98
Crude Fat (or Ether Extract)	%	3.21	4.92	3.73
Linoleic acid	%	1.64	2.61	2.01
Crude Fiber	%	4.32	5.41	5.12
Crude Ash	%	3.41	3.10	3.07
Nitrogen-Free Extract	%	52.15	50.82	55.69
Starch	%	34.32	34.74	38.72
Sugar/Total Sugars	%	3.30	2.83	2.79
Nitrogen-free residue	%	18.81	18.63	19.26
Amino acids, gross content				
Lysine	%	1.28	1.03	0.97
Methionine	%	0.63	0.61	0.58
Methionine + Cystine	%	0.96	0.93	0.87
Threonine	%	0.79	0.70	0.64
Tryptophan	%	0.28	0.25	0.24
Arginine	%	1.35	1.25	1.13
Valine	%	1.03	0.96	0.91
Histidine	%	0.55	0.50	0.46
Glycine	%	0.97	0.92	0.84
Isoleucine	%	0.96	0.87	0.81
Leucine	%	1.48	1.36	1.25
Phenylalanine	%	1.02	0.91	0.86
Tyrosine	%	0.71	0.62	0.58
Macronutrients				
Calcium	%	1.04	0.95	0.95
Total Phosphorus	%	0.74	0.71	0.74
Available Phosphorus	%	0.44	0.41	0.43
Potassium	%	0.83	0.73	0.75
Sodium	%	0.13	0.11	0.11
Chlorine/Chloride	%	0.22	0.21	0.21
Vitamins and trace elements				
Vitamin A	million IU/t	12.00	12.00	12.00
Vitamin D3	million IU/t	4.00	4.00	4.00
Vitamin E	%	30.00	30.00	30.00
Vitamin K	%	4.00	4.00	4.00
Vitamin B1 (Thiamine)	%	4.00	4.00	4.00
Fe	%	40.00	40.00	40.00
Cu	%	20.00	20.00	20.00
Zn	%	100.00	100.00	100.00
Mn	%	120.00	120.00	120.00
J	%	1.00	1.00	1.00
Se	%	0.30	0.30	0.30

**Table 3:** Stocking density standards for broilers Smena 9 in battery cages [33].

Target Live Weight at Slaughter, kg	Poultry Stocking Density	
	Floor Area per Bird (cm^2^)	Recommended Stocking Density (Birds/m^2^)
1.1–1.2	265	38
1.3–1.4	312	32
1.5–1.6	357	28
1.7–1.8	385	26
1.9–2.0	435	23
2.1–2.2	476	21
2.3–2.6	570	17

**Table 4:** Temperature and humidity conditions for broilers in cages.

Age (Weeks)	Room Temperature (°C)	Relative Humidity (%)
1	33–31	40–60
2–3	20–23	60–70
4–6	20–23	60–70
7	18–19	60–70

**Table 5:** Broiler meat amino acid composition at the age of 24 days, g/100 g (M ± SEM, n = 10).

Item	Group				*p*-Value
	S(-)CON	S(+)CON	S(+)DHQEC_21	S(+)DHQEC_1	
Breast Meat					
ASP *	8.88 ± 0.08	8.63 ± 0.12	8.26 ± 0.22	8.52 ± 0.16	0.06
THR	4.04 ± 0.08	4.09 ± 0.06	3.94 ± 0.10	3.95 ± 0.05	0.44
SER	3.22 ± 0.04	3.33 ± 0.07	3.23 ± 0.08	3.24 ± 0.04	0.52
GLU *	12.92 ± 0.25	13.04 ± 0.23	12.34 ± 0.36	12.97 ± 0.21	0.26
GLY *	3.40 ± 0.08	3.41 ± 0.04	3.24 ± 0.08	3.21 ± 0.06	0.07
ALA *	5.81 ± 0.09	5.87 ± 0.07	5.66 ± 0.12	5.70 ± 0.07	0.32
CYS	0.88 ± 0.01	0.87 ± 0.02	0.86 ± 0.01	0.86 ± 0.01	0.58
VAL	4.86 ± 0.07	4.85 ± 0.07	4.71 ± 0.12	4.73 ± 0.06	0.38
MET	1.81 ± 0.04	1.77 ± 0.04	1.75 ± 0.03	1.76 ± 0.03	0.59
ILE	4.77 ± 0.07	4.32 ± 0.09	4.67 ± 0.11	4.67 ± 0.06	0.63
LEU	7.27 ± 0.11	7.32 ± 0.09	7.07 ± 0.16	7.12 ± 0.10	0.36
TYR	3.05 ± 0.05	3.10 ± 0.05	3.04 ± 0.07	2.91 ± 0.13	0.43
PHE	3.73 ± 0.05	3.79 ± 0.05	3.67 ± 0.08	3.68 ± 0.04	0.40
HIS	3.06 ± 0.05	3.10 ± 0.08	3.08 ± 0.11	3.05 ± 0.05	0.96
LYS	8.36 ± 0.13	8.40 ± 0.12	8.11 ± 0.19	8.02 ± 0.12	0.17
ARG *	6.35 ± 0.12	6.42 ± 0.13	6.44 ± 0.15	6.21 ± 0.09	0.50
PRO	2.66 ± 0.08 ^c^	2.64 ± 0.11 ^c^	2.27 ± 0.10 ^ab^	2.57 ± 0.08	0.03
SAA	85.09 ± 1.08	85.39 ± 1.00	82.36 ± 1.86	83.16 ± 1.00	0.26
SEAA	39.95 ± 0.52	40.02 ± 0.52	38.04 ± 0.92	39.12 ± 0.55	0.13
SNEAA	37.91 ± 0.55	38.09 ± 0.48	37.01 ± 0.84	36.96 ± 0.46	0.39
SFAA *	37.36 ± 0.47	37.36 ± 0.46	35.94 ± 0.87	36.61 ± 0.46	0.27
Thigh meat					
ASP *	7.01 ± 0.18	6.56 ± 0.18	6.67 ± 0.16	6.27 ± 0.20	0.05
THR	3.49 ± 0.08 ^d^	3.29 ± 0.09	3.21 ± 0.08	3.14 ± 0.09 ^a^	0.03
SER	2.92 ± 0.09	2.73 ± 0.09	2.68 ± 0.06	2.68 ± 0.09	0.17
GLU *	11.22 ± 0.34	10.72 ± 0.31	10.80 ± 0.29	10.26 ± 0.35	0.21
GLY *	3.26 ± 0.09	3.35 ± 0.16	3.03 ± 0.06	2.99 ± 0.10	0.07
ALA *	5.06 ± 0.12 ^d^	4.86 ± 0.12	4.70 ± 0.10	4.53 ± 0.13 ^a^	0.02
CYS	0.79 ± 0.02 ^d^	0.74 ± 0.02	0.77 ± 0.02	0.71 ± 0.03 ^a^	0.04
VAL	3.97 ± 0.08 ^d^	3.73 ± 0.09	3.68 ± 0.08	3.60 ± 0.11 ^a^	0.05
MET	1.60 ± 0.03 ^d^	1.50 ± 0.04	1.48 ± 0.02	1.43 ± 0.05 ^a^	0.03
ILE	3.96 ± 0.07 ^d^	3.67 ± 0.10	3.70 ± 0.08	3.58 ± 0.10 ^a^	0.03
LEU	6.20 ± 0.13 ^d^	5.82 ± 0.15	5.75 ± 0.11	5.60 ± 0.16 ^a^	0.02
TYR	2.61 ± 0.07	2.44 ± 0.07	2.44 ± 0.06	2.38 ± 0.07	0.02
PHE	3.29 ± 0.07 ^d^	3.04 ± 0.08	3.06 ± 0.06	2.96 ± 0.08 ^a^	0.12
HIS	2.04 ± 0.04 ^d^	1.92 ± 0.05	1.94 ± 0.04	1.87 ± 0.04 ^a^	0.03
LYS	6.99 ± 0.16 ^d^	6.51 ± 0.20	6.39 ± 0.13	6.29 ± 0.22 ^a^	0.06
ARG *	5.65 ± 0.17	5.26 ± 0.17	5.42 ± 0.12	5.14 ± 0.14	0.12
PRO	2.58 ± 0.08	2.60 ± 0.11	2.22 ± 0.12	2.32 ± 0.13	0.06
SAA	72.66 ± 1.65 ^d^	68.77 ± 1.76	67.95 ± 1.45	65.76 ± 1.92 ^a^	0.05
SEAA	34.68 ± 0.91	33.27 ± 0.90	32.54 ± 0.79	31.43 ± 0.97	0.09
SNEAA	31.55 ± 0.63	29.50 ± 0.76	29.22 ± 0.56	28.48 ± 0.83	0.03
SFAA *	32.21 ± 0.82 ^dd^	30.75 ± 0.83	30.62 ± 0.69	29.19 ± 0.87 ^aa^	0.09

Notes here and below: Significant by Tukey’s test with Group 1 (control): ^a^—*p* < 0.05, ^aa^—*p* < 0.01. Significant by Tukey’s test with Group 2: ^b^—*p* < 0.05. Significant by Tukey’s test with Group 3: ^c^—*p* < 0.05. Significant by Tukey’s test with Group 4: ^d^—*p* < 0.05, ^dd^—*p* < 0.01. S(-)CON, recommended stocking density for this cross. S(+)CON—the stocking density was increased by 10% starting day 21 of the experiment. S(+)DHQEC_21—the stocking density was increased by 10% from day 21 of the experiment + DHQEC from day 21 of the experiment. S(+)DHQEC_1—the stocking density was increased by 10% from day 21 of the experiment + DHQEC from day 1 of the experiment. SAA—Sum of amino acids. SEAA—Sum of essential amino acids. SNEAA-Sum of nonessential amino acids. SFAA—Sum of flavor amino acids *.

**Table 6:** Broiler meat amino acid composition at the age of 34 days, g/100 g (M ± SEM, n = 10).

Item	Group				*p*-Value
	S(-)CON	S(+)CON	S(+)DHQEC_21	S(+)DHQEC_1	
Breast meat					
ASP *	9.45 ± 0.10	9.53 ± 0.10	8.69 ± 0.17	8.40 ± 0.12	<0.001
THR	4.06 ± 0.04	4.21 ± 0.08 ^cd^	3.92 ± 0.05 ^b^	3.83 ± 0.07 ^b^	0.002
SER	3.32 ± 0.03	3.48 ± 0.08	3.27 ± 0.07	3.35 ± 0.08	0.15
GLU *	14.25 ± 0.20 ^cddd^	14.10 ± 0.05 ^dd^	13.64 ± 0.14 ^a^	13.16 ± 0.07 ^aaabb^	<0.001
GLY *	3.32 ± 0.02 ^cccddd^	3.49 ± 0.04 ^ccddd^	3.78 ± 0.07 ^aaabb^	3.88 ± 0.08 ^aaabbb^	<0.001
ALA *	6.20 ± 0.17 ^dd^	6.07 ± 0.10 ^d^	5.72 ± 0.13	5.42 ± 0.12 ^aab^	0.04
CYS	0.88 ± 0.02	0.92 ± 0.02	0.90 ± 0.02	0.86 ± 0.02	0.30
VAL	4.85 ± 0.06 ^dd^	4.87 ± 0.12 ^dd^	4.51 ± 0.10	4.27 ± 0.10 ^aabb^	<0.001
MET	1.90 ± 0.03 ^cccddd^	1.88 ± 0.04 ^cccddd^	2.30 ± 0.11 ^aaabbb^	2.38 ± 0.06 ^aaabbb^	<0.001
ILE	4.75 ± 0.07 ^dd^	4.75 ± 0.11 ^dd^	4.45 ± 0.08	4.28 ± 0.10 ^aabb^	0.0030
LEU	7.67 ± 0.19	7.60 ± 0.10	7.75 ± 0.13	7.54 ± 0.16	0.86
TYR	3.16 ± 0.05 ^bcccd^	3.34 ± 0.05 ^acc^	3.64 ± 0.04 ^aaabbd^	3.41 ± 0.06 ^ac^	0.003
PHE	3.79 ± 0.05 ^cdd^	3.89 ± 0.06	4.16 ± 0.16 ^a^	4.24 ± 0.09 ^aa^	<0.001
HIS	3.19 ± 0.12	3.14 ± 0.09	3.64 ± 0.16	3.42 ± 0.11	0.04
LYS	8.71 ± 0.19	8.73 ± 0.24	8.56 ± 0.15	8.12 ± 0.11	0.17
ARG*	6.62 ± 0.14	6.47 ± 0.21	6.65 ± 0.46	7.00 ± 0.18	0.52
PRO	2.84 ± 0.09 ^bb^	3.47 ± 0.07 ^aadd^	3.27 ± 0.28	2.71 ± 0.13 ^bb^	0.003
SAA	88.97 ± 0.90	89.94 ± 0.68	88.85 ± 0.81	86.29 ± 1.26	0.08
SEAA	42.54 ± 0.41 ^ddd^	43.48 ± 0.24 ^ddd^	42.02 ± 0.54	40.33 ± 0.48 ^aaabbb^	<0.001
SNEAA	38.92 ± 0.49	39.07 ± 0.44	39.29 ± 0.67	38.10 ± 0.78	0.58
SFAA *	39.85 ± 0.43	39.66 ± 0.20	38.48 ± 0.27	37.86 ± 0.45	0.003
Thigh meat					
ASP*	7.07 ± 0.19	7.10 ± 0.14	6.68 ± 0.15	6.43 ± 0.19	0.03
THR	3.47 ± 0.08	3.38 ± 0.09	3.18 ± 0.06	3.14 ± 0.08	0.02
SER	2.92 ± 0.06	2.85 ± 0.10	2.70 ± 0.06	2.79 ± 0.07	0.25
GLU *	11.80 ± 0.36	11.42 ± 0.22	11.19 ± 0.20	11.02 ± 0.29	0.28
GLY *	3.09 ± 0.09 ^ccd^	3.08 ± 0.07 ^ccd^	3.62 ± 0.11 ^aabb^	3.57 ± 0.18 ^ab^	<0.001
ALA *	5.05 ± 0.14	4.89 ± 0.12	4.76 ± 0.14	4.58 ± 0.15	0.17
CYS	0.76 ± 0.02	0.75 ± 0.02	0.72 ± 0.02	0.75 ± 0.02	0.64
VAL	3.97 ± 0.09 ^cdd^	3.79 ± 0.10	3.57 ± 0.09 ^a^	3.41 ± 0.09 ^aa^	0.003
MET	1.63 ± 0.02 ^cccddd^	1.56 ± 0.01 ^cccddd^	1.87 ± 0.08 ^aabbbd^	2.08 ± 0.05 ^aaabbbc^	<0.001
ILE	3.99 ± 0.09 ^ccdd^	3.79 ± 0.10	3.50 ± 0.06 ^aa^	3.44 ± 0.08 ^aa^	<0.001
LEU	6.28 ± 0.14	6.10 ± 0.13	6.19 ± 0.12	6.22 ± 0.16	0.82
TYR	2.69 ± 0.07	2.72 ± 0.03	2.84 ± 0.04	2.87 ± 0.09	0.04
PHE	3.31 ± 0.07	3.20 ± 0.07 ^d^	3.40 ± 0.10	3.58 ± 0.09 ^b^	0.11
HIS	2.12 ± 0.05 ^a^	1.96 ± 0.05 ^ccddd^	2.25 ± 0.08 ^bb^	2.38 ± 0.07 ^abbb^	0.0002
LYS	7.39 ± 0.20 ^b^	6.72 ± 0.16 ^a^	6.79 ± 0.15	6.69 ± 0.15	0.022
ARG *	5.67 ± 0.13	5.07 ± 0.19	5.13 ± 0.28	5.80 ± 0.23	0.05
PRO	2.55 ± 0.11 ^cc^	2.89 ± 0.06	3.18 ± 0.22 ^aadd^	2.38 ± 0.08 ^cc^	0.002
SAA	73.77 ± 1.61	71.30 ± 1.46	71.57 ± 0.17	71.14 ± 1.95	0.58
SEAA	35.17 ± 0.91	34.97 ± 0.61	34.98 ± 0.64	33.64 ± 0.98	0.61
SNEAA	32.17 ± 0.67	30.52 ± 0.67	30.74 ± 0.56	30.95 ± 0.75	0.27
SFAA *	32.67 ± 0.78	31.56 ± 0.69	31.39 ± 0.56	31.40 ± 1.00	0.55

Notes here and below: Significant by Tukey’s test with Group 1 (control): ^a^—*p* < 0.05, ^aa^—*p* < 0.01, ^aaa^—*p* < 0.001. Significant by Tukey’s test with Group 2: ^b^—*p* < 0.05, ^bb^—*p* < 0.01, ^bbb^—*p* < 0.001. Significant by Tukey’s test with Group 3: ^c^—*p* < 0.05, ^cc^—*p* < 0.01, ^ccc^—*p* < 0.001. Significant by Tukey’s test with Group 4: ^d^—*p* < 0.05, ^dd^—*p* < 0.01, ^ddd^—*p* < 0.001. S(-)CON, recommended stocking density for this cross. S(+)CON—the stocking density was increased by 10% starting day 21 of the experiment. S(+)DHQEC_21—the stocking density was increased by 10% from day 21 of the experiment + DHQEC from day 21 of the experiment. S(+)DHQEC_1—the stocking density was increased by 10% from day 21 of the experiment + DHQEC from day 1 of the experiment. SAA—Sum of amino acids. SEAA—Sum of essential amino acids. SNEAA—Sum of nonessential amino acids. SFAA—Sum of flavor amino acids *.

**Table 7:** Broiler meat amino acid composition at the age of 52 days, g/100 g (M ± SEM, n = 10).

Item	Group				*p*-Value
	S(-)CON	S(+)CON	S(+)DHQEC_21	S(+)DHQEC_1	
Breast meat					
ASP *	8.43 ± 0.06	8.36 ± 0.13	8.38 ± 0.12	8.57 ± 0.11	0.56
THR	3.95 ± 0.04	3.88 ± 0.03	3.82 ± 0.05	3.82 ± 0.10	0.33
SER	3.52 ± 0.05 ^cdd^	3.44 ± 0.04	3.31 ± 0.02 ^b^	3.26 ± 0.09 ^bb^	0.01
GLU *	16.67 ± 0.12	13.34 ± 0.17	13.26 ± 0.11	13.25 ± 0.14	0.17
GLY *	3.89 ± 0.07	3.69 ± 0.05 ^dd^	3.73 ± 0.05 ^d^	4.01 ± 0.10 ^bbc^	0.01
ALA *	5.54 ± 0.06 ^dd^	5.44 ± 0.07 ^ddd^	5.40 ± 0.04 ^ddd^	5.87 ± 0.10 ^aabbbccc^	<0.001
CYS	0.81 ± 0.02	0.82 ± 0.02	0.89 ± 0.01	0.82 ± 0.03	0.04
VAL	4.33 ± 0.05	4.39 ± 0.08	4.37 ± 0.09	4.54 ± 0.08	0.31
MET	2.17 ± 0.05 ^bcccddd^	2.39 ± 0.04 ^a^	2.49 ± 0.02 ^aaa^	2.53 ± 0.08 ^aaa^	<0.001
ILE	4.27 ± 0.05 ^d^	4.31 ± 0.04 ^d^	4.36 ± 0.10	4.61 ± 0.08 ^ab^	0.02
LEU	7.70 ± 0.06	7.61 ± 0.09	7.58 ± 0.06	7.83 ± 0.19	0.37
TYR	3.46 ± 0.05 ^ccc^	3.46 ± 0.04 ^ccc^	4.36 ± 0.19 ^aaabbb^	3.91 ± 0.13	<0.001
PHE	4.16 ± 0.05	4.18 ± 0.04	4.17 ± 0.09	4.11 ± 0.04	0.86
HIS	3.53 ± 0.12	3.64 ± 0.09	3.87 ± 0.08	4.07 ± 0.19	0.03
LYS	8.92 ± 0.19	8.65 ± 0.16	8.49 ± 0.08	8.99 ± 0.19	0.10
ARG *	6.02 ± 0.09	6.26 ± 0.20	6.52 ± 0.30	5.59 ± 0.28	0.07
PRO	3.22 ± 0.09	3.10 ± 0.14	3.29 ± 0.12	2.93 ± 0.16	0.30
SAA	87.59 ± 0.76	86.94 ± 0.86	88.27 ± 0.84	88.71 ± 0.50	0.40
SEAA	41.73 ± 0.40	40.83 ± 0.56	41.73 ± 0.29	41.80 ± 0.37	0.31
SNEAA	39.04 ± 0.35	39.03 ± 0.41	39.13 ± 0.41	40.50 ± 0.34	0.04
SFAA *	37.55 ± 0.33	37.10 ± 0.32	37.29 ± 0.37	37.29 ± 0.17	0.80
Thigh meat					
ASP *	7.42 ± 0.17	6.83 ± 0.24	6.90 ± 0.16	7.05 ± 0.10	0.13
THR	3.44 ± 0.09	3.19 ± 0.12	3.26 ± 0.08	3.27 ± 0.04	0.28
SER	3.09 ± 0.07	2.88 ± 0.11	2.88 ± 0.09	2.74 ± 0.05	0.08
GLU *	12.56 ± 0.23	11.52 ± 0.38	11.97 ± 0.29	11.78 ± 0.09	0.09
GLY *	4.03 ± 0.11	3.69 ± 0.11 ^d^	3.59 ± 0.07 ^dd^	4.21 ± 0.21 ^bcc^	0.01
ALA *	5.02 ± 0.09	4.61 ± 0.14 ^dd^	4.65 ± 0.10 ^d^	5.15 ± 0.08 ^bbc^	0.004
CYS	0.76 ± 0.003	0.72 ± 0.02	0.77 ± 0.02	0.74 ± 0.02	0.23
VAL	3.70 ± 0.09	3.48 ± 0.12	3.58 ± 0.08	3.68 ± 0.05	0.32
MET	2.06 ± 0.04	2.06 ± 0.07	2.13 ± 0.06	2.17 ± 0.02	0.43
ILE	3.71 ± 0.10	3.53 ± 0.12	3.62 ± 0.09	3.85 ± 0.04	0.16
LEU	6.73 ± 0.15	6.30 ± 0.21	6.37 ± 0.14	6.80 ± 0.08	0.09
TYR	3.06 ± 0.12	2.95 ± 0.11 ^c^	3.49 ± 0.12 ^b^	3.19 ± 0.14	0.03
PHE	3.70 ± 0.10	3.54 ± 0.13	3.58 ± 0.08	3.60 ± 0.04	0.70
HIS	2.54 ± 0.10	2.40 ± 0.10	2.50 ± 0.09	2.56 ± 0.05	0.56
LYS	7.54 ± 0.21	7.19 ± 0.25	7.13 ± 0.17	7.59 ± 0.13	0.27
ARG *	5.42 ± 0.20	5.56 ± 0.21	5.56 ± 0.26	5.00 ± 0.16	0.24
PRO	3.01 ± 0.08	2.65 ± 0.09	2.87 ± 0.16	2.78 ± 0.12	0.22
SAA	77.80 ± 1.71	73.10 ± 2.27	74.85 ± 1.47	76.14 ± 0.58	0.27
SEAA	38.20 ± 0.77	35.14 ± 1.09	36.35 ± 0.67	36.88 ± 0.39	0.08
SNEAA	33.42 ± 0.80	31.68 ± 1.06	32.17 ± 0.72	33.52 ± 0.31	0.29
SFAA *	34.45 ± 0.69	32.21 ± 0.97	32.68 ± 0.65	33.18 ± 0.22	0.18

Notes here and below: Significant by Tukey’s test with Group 1 (control): ^a^—*p* < 0.05, ^aa^—*p* < 0.01, ^aaa^—*p* < 0.001. Significant by Tukey’s test with Group 2: ^b^—*p* < 0.05, ^bb^—*p* < 0.01, ^bbb^—*p* < 0.001. Significant by Tukey’s test with Group 3: ^c^—*p* < 0.05, ^cc^—*p* < 0.01, ^ccc^—*p* < 0.001. Significant by Tukey’s test with Group 4: ^d^—*p* < 0.05, ^dd^—*p* < 0.01, ^ddd^—*p* < 0.001. S(-)CON, recommended stocking density for this cross. S(+)CON—the stocking density was increased by 10% starting day 21 of the experiment. S(+)DHQEC_21—the stocking density was increased by 10% from day 21 of the experiment + DHQEC from day 21 of the experiment. S(+)DHQEC_1—the stocking density was increased by 10% from day 21 of the experiment + DHQEC from day 1 of the experiment. SAA—Sum of amino acids. SEAA—Sum of essential amino acids. SNEAA—Sum of nonessential amino acids. SFAA—Sum of flavor amino acids *.

**Table 8:** The “age” factor had an uneven effect on the amino acid composition of poultry meat in the different groups (n = 10).

Item	Group							
	S(-)CON		S(+)CON		S(+)DHQEC_21		S(+)DHQEC_1	
	F	*p*	F	*p*	F	*p*	F	*p*
Breast meat								
ASP *	30.050	≤0.001	24.420	≤0.001	1.330	0.288	0.270	0.764
THR	0.710	0.501	5.690	0.010	0.760	0.483	1.260	0.304
SER	12.530	≤0.001	1.240	0.308	0.370	0.695	0.920	0.413
GLU *	10.370	0.001	8.920	0.001	6.360	0.008	0.650	0.532
GLY *	20.830	≤0.001	12.090	≤0.001	19.340	≤0.001	35.820	<0.001
ALA *	6.030	0.008	14.100	≤0.001	2.340	0.124	4.790	0.020
CYS	4.260	0.027	7.310	0.004	2.170	0.141	1.120	0.346
VAL	18.070	≤0.001	8.190	0.002	2.750	0.089	8.750	0.002
MET	20.300	≤0.001	66.100	≤0.001	48.490	≤0.001	74.100	<0.001
ILE	12.780	≤0.001	9.750	0.001	2.610	0.100	6.670	0.006
LEU	2.710	0.088	3.150	0.063	7.560	0.004	7.360	0.004
TYR	14.500	≤0.001	15.290	≤0.001	35.150	≤0.001	15.270	<0.001
PHE	16.420	≤0.001	15.920	≤0.001	7.600	0.004	27.140	<0.001
HIS	4.730	0.019	11.390	≤0.001	13.320	≤0.001	23.000	<0.001
LYS	2.480	0.106	1.060	0.364	2.430	0.115	13.230	<0.001
ARG *	4.950	0.016	0.350	0.710	0.120	0.886	14.380	<0.001
PRO	8.070	0.002	15.480	≤0.001	13.690	0.001	2.490	0.108
SAA	4.520	0.022	6.910	0.005	6.530	0.007	7.670	0.003
SEAA	8.960	0.001	15.180	≤0.001	10.090	0.001	6.350	0.007
SNEAA	1.510	0.243	1.560	0.232	3.430	0.053	10.810	0.001
SFAA *	10.420	0.001	13.550	≤0.001	3.600	0.047	2.120	0.146
Thigh meat								
ASP *	1.110	0.345	2.620	0.092	0.590	0.560	4.200	0.030
THR	0.090	0.914	0.960	0.396	0.260	0.777	0.620	0.546
SER	1.380	0.272	0.590	0.561	2.160	0.139	0.480	0.623
GLU *	3.280	0.056	2.210	0.131	5.130	0.015	5.730	0.011
GLY *	22.450	≤0.001	6.060	0.007	15.290	≤0.001	17.580	<0.001
ALA	0.030	0.969	1.200	0.319	0.200	0.817	5.950	0.009
CYS	1.400	0.266	0.690	0.511	1.780	0.192	0.740	0.488
VAL	2.310	0.122	2.260	0.125	0.560	0.577	1.460	0.255
MET	55.120	≤0.001	53.830	≤0.001	31.110	0.000	74.980	<0.001
ILE	2.530	0.102	1.430	0.259	1.760	0.196	3.710	0.043
LEU	3.300	0.055	2.200	0.131	6.490	0.006	15.250	<0.001
TYR	7.140	0.004	12.910	≤0.001	47.290	≤0.001	18.930	<0.001
PHE	6.970	0.004	6.630	0.005	10.370	0.001	23.150	<0.001
HIS	17.340	≤0.001	16.590	≤0.001	16.050	≤0.001	55.540	<0.001
LYS	2.100	0.145	2.640	0.091	5.810	0.009	9.880	0.001
ARG *	0.540	0.590	1.510	0.241	0.930	0.408	5.080	0.016
PRO	5.730	0.010	3.310	0.053	8.370	0.002	3.350	0.055
SAA	2.090	0.146	1.390	0.267	6.100	0.008	8.110	0.003
SEAA	3.500	0.047	1.550	0.233	7.110	0.004	8.530	0.002
SNEAA	1.600	0.224	1.650	0.213	5.700	0.010	10.490	0.001
SFAA *	1.740	0.198	0.730	0.491	2.480	0.107	5.640	0.011

Notes here and below: S(-)CON, recommended stocking density for this cross. S(+)CON—the stocking density was increased by 10% starting day 21 of the experiment. S(+)DHQEC_21—the stocking density was increased by 10% from day 21 of the experiment + DHQEC from day 21 of the experiment. S(+)DHQEC_1—the stocking density was increased by 10% from day 21 of the experiment + DHQEC from day 1 of the experiment. SAA—Sum of amino acids. SEAA—Sum of essential amino acids. SNEAA—Sum of nonessential amino acids. F—Fisher’s criterion. *p*—significance level. SFAA—Sum of flavor amino acids *.

**Table 9:** The influence of the “sex” factor on the broiler muscle tissue amino acid composition (n = 40).

Item	Age, Days					
	24		34		52	
	F	*p*	F	*p*	F	*p*
Breast meat						
ASP *	2.950	0.090	0.236	0.631	0.140	0.710
THR	4.890	0.030	0.781	0.384	0.000	0.990
SER	2.700	0.110	0.103	0.750	1.600	0.220
GLU *	5.270	0.030	0.144	0.707	0.070	0.800
GLY *	2.890	0.100	0.086	0.772	5.500	0.030
ALA *	5.530	0.020	0.007	0.933	0.460	0.500
CYS	0.100	0.750	0.026	0.873	0.610	0.440
VAL	5.270	0.030	0.254	0.618	1.590	0.220
MET	0.820	0.370	0.123	0.729	0.370	0.550
ILE	6.240	0.020	0.339	0.565	0.850	0.370
LEU	5.520	0.020	0.227	0.638	0.380	0.540
TYR	0.090	0.770	0.529	0.473	0.040	0.840
PHE	5.630	0.020	0.035	0.854	1.480	0.240
HIS	4.840	0.030	0.089	0.767	0.730	0.400
LYS	2.680	0.110	0.562	0.460	0.100	0.760
ARG *	1.260	0.270	0.030	0.865	3.260	0.080
PRO	0.220	0.640	0.621	0.437	3.800	0.060
SAA	5.400	0.030	0.013	0.911	0.070	0.800
SEAA	4.570	0.040	0.432	0.516	2.130	0.160
SNEAA	5.550	0.020	0.143	0.709	0.610	0.440
SFAA *	5.580	0.020	0.109	0.744	0.180	0.680
Thigh meat						
ASP *	0.010	0.920	1.160	0.290	2.490	0.130
THR	0.070	0.790	1.800	0.190	1.570	0.220
SER	0.010	0.910	2.020	0.160	2.130	0.160
GLU *	0.001	0.980	4.510	0.040	4.070	0.060
GLY *	1.260	0.270	0.260	0.610	5.240	0.030
ALA *	0.250	0.620	0.750	0.390	3.830	0.060
CYS	0.010	0.920	1.820	0.190	0.060	0.820
VAL	0.000	0.990	0.380	0.540	3.910	0.060
MET	0.120	0.730	0.110	0.740	0.540	0.470
ILE	0.000	0.980	0.530	0.470	1.840	0.190
LEU	0.020	0.890	1.890	0.180	4.700	0.040
TYR	0.010	0.910	1.810	0.190	0.590	0.450
PHE	0.000	0.950	1.110	0.300	2.750	0.110
HIS	0.020	0.880	0.050	0.830	5.420	0.030
LYS	0.110	0.740	0.820	0.370	10.240	0.004
ARG *	0.580	0.450	0.130	0.720	0.040	0.850
PRO	0.100	0.760	0.040	0.840	7.330	0.010
SAA	0.020	0.890	1.900	0.180	5.610	0.030
SEAA	0.060	0.820	2.520	0.120	6.550	0.020
SNEAA	0.010	0.920	1.290	0.260	5.180	0.030
SFAA *	0.130	0.720	2.080	0.160	4.600	0.040

Notes here and below: F—Fisher’s criterion. *p*—significance level. SFAA—Sum of flavor amino acids *.

**Table 10:** Effect of the “tissue” factor on the broiler muscle amino acid composition (n = 120).

Item	Age, Days					
	24		34		52	
	F	*p*	F	*p*	F	*p*
ASP *	237.500	≤0.001	261.720	≤0.001	160.980	<0.001
THR	153.630	≤0.001	140.100	≤0.001	112.190	<0.001
SER	92.700	≤0.001	101.490	≤0.001	69.370	<0.001
GLU *	97.500	≤0.001	181.890	≤0.001	73.500	<0.001
GLY *	5.700	0.019	10.500	0.002	0.200	0.660
ALA *	147.930	≤0.001	92.060	≤0.001	71.400	<0.001
CYS	82.140	≤0.001	102.230	≤0.001	32.040	<0.001
VAL	268.740	≤0.001	121.920	≤0.001	165.140	<0.001
MET	101.220	≤0.001	28.270	≤0.001	42.930	<0.001
ILE	253.260	≤0.001	120.290	≤0.001	115.980	<0.001
LEU	202.030	≤0.001	203.900	≤0.001	125.350	<0.001
TYR	94.780	≤0.001	144.500	≤0.001	28.250	<0.001
PHE	164.270	≤0.001	82.250	≤0.001	102.950	<0.001
HIS	708.050	≤0.001	247.740	≤0.001	239.440	<0.001
LYS	183.760	≤0.001	132.990	≤0.001	112.750	<0.001
ARG *	97.540	≤0.001	60.490	≤0.001	17.630	<0.001
PRO	1.970	0.164	5.580	0.021	11.500	0.001
SAA	186.560	≤0.001	309.700	0.000	174.870	<0.001
SEAA	121.200	≤0.001	229.540	≤0.001	104.170	<0.001
SNEAA	256.100	≤0.001	293.440	≤0.001	215.470	<0.001
SFAA *	142.430	≤0.001	258.320	≤0.001	109.730	<0.001

Notes here and below: F—Fisher’s criterion. *p*—significance level. SFAA—Sum of flavor amino acids *.

## Data Availability

The original data presented in this study are openly available in the GitHub 3.6.2 repository at https://github.com/Bogolyubovajulia/Amino-acid-composition-of-chicken-meat (accessed on 30 May 2026). This study was conducted in accordance with the research and publication ethics guidelines of MDPI (https://www.mdpi.com/ethics, accessed on 30 May 2026).

## Abbreviations

The following abbreviations are used in this manuscript:

ASP	Aspartic acid
THR	Threonine
SER	Serine
GLU	Glutamic acid
GLY	Glycine
ALA	Alanine
VAL	Valine
ILE	Isoleucine
LEU	Leucine
TYR	Tyrosine
PHE	Phenylalanine
HIS	Histidine
LYS	Lysine
ARG	Arginine
PRO	Proline
SAA	Sum of amino acids
SEAA	Sum of essential amino acids
SNEAA	Sum of nonessential amino acids
SFAA	Sum of flavor amino acids
DHQ	Dihydroquercetin
EAA	Essential Amino Acids
DM	Dry Matter
ANOVA	Analysis of Variance
AA	Amino Acids
